# Engineering towards a complete heterologous cellulase secretome in *Yarrowia lipolytica* reveals its potential for consolidated bioprocessing

**DOI:** 10.1186/s13068-014-0148-0

**Published:** 2014-10-16

**Authors:** Hui Wei, Wei Wang, Markus Alahuhta, Todd Vander Wall, John O Baker, Larry E Taylor, Stephen R Decker, Michael E Himmel, Min Zhang

**Affiliations:** Biosciences Center, National Renewable Energy Laboratory, Golden, CO 80401 USA; National Bioenergy Center, National Renewable Energy Laboratory, Golden, CO 80401 USA

**Keywords:** *Yarrowia lipolytica*, Oleaginous yeast, Heterologous expression, Cellulase, Cellobiohydrolase I, Cellobiohydrolase II, Endoglucanase II, Cell consortia, Advanced biofuels

## Abstract

**Background:**

*Yarrowia lipolytica* is an oleaginous yeast capable of metabolizing glucose to lipids, which then accumulate intracellularly. However, it lacks the suite of cellulolytic enzymes required to break down biomass cellulose and cannot therefore utilize biomass directly as a carbon source. Toward the development of a direct microbial conversion platform for the production of hydrocarbon fuels from cellulosic biomass, the potential for *Y. lipolytica* to function as a consolidated bioprocessing strain was investigated by first conducting a genomic search and functional testing of its endogenous glycoside hydrolases. Once the range of endogenous enzymes was determined, the critical cellulases from *Trichoderma reesei* were cloned into *Yarrowia.*

**Results:**

Initially, work to express *T. reesei* endoglucanase II (EGII) and cellobiohydrolase (CBH) II in *Y. lipolytica* resulted in the successful secretion of active enzymes. However, a critical cellulase, *T. reesei* CBHI, while successfully expressed in and secreted from *Yarrowia*, showed less than expected enzymatic activity, suggesting an incompatibility (probably at the post-translational level) for its expression in *Yarrowia*. This result prompted us to evaluate alternative or modified CBHI enzymes. Our subsequent expression of a *T. reesei-Talaromyces emersonii* (Tr-Te) chimeric CBHI, *Chaetomium thermophilum* CBHI, and *Humicola grisea* CBHI demonstrated remarkably improved enzymatic activities. Specifically, the purified chimeric Tr-Te CBHI showed a specific activity on Avicel that is comparable to that of the native *T. reesei* CBHI. Furthermore, the chimeric Tr-Te CBHI also showed significant synergism with EGII and CBHII in degrading cellulosic substrates, using either mixed supernatants or co-cultures of the corresponding *Y. lipolytica* transformants. The consortia system approach also allows rational volume mixing of the transformant cultures in accordance with the optimal ratio of cellulases required for efficient degradation of cellulosic substrates.

**Conclusions:**

Taken together, this work demonstrates the first case of successful expression of a chimeric CBHI with essentially full native activity in *Y. lipolytica*, and supports the notion that *Y. lipolytica* strains can be genetically engineered, ultimately by heterologous expression of fungal cellulases and other enzymes, to directly convert lignocellulosic substrates to biofuels.

**Electronic supplementary material:**

The online version of this article (doi:10.1186/s13068-014-0148-0) contains supplementary material, which is available to authorized users.

## Background

The accumulation of lipids by oleaginous microorganisms can be exploited as a route to biodiesel production. So far, however, only sugars and agro-industrial wastes have been used to culture these microorganisms, and the use of these carbon sources inevitably increases the cost of biofuel production. Recently, *Yarrowia lipolytica* has become one of the model oleaginous yeasts for the development of biofuels [1-3].

The following features make *Y. lipolytica* a unique model in developing biofuels. First, *Y. lipolytica* is known as an oleaginous microorganism that intracellularly accumulates lipids, which could serve as an alternative to plant oils for biodiesel production [4]. Secondly, among the “non-conventional” yeasts, *Y. lipolytica* is one of the most extensively studied model organisms for its genetic and physiological properties, as well as for its biotechnological applications [5]. The availability of the genome sequence of *Y. lipolytica* strain E150 (CLIB99) [6,7] and the development of genetic tools, such as transformation methods [8], and integrative expression cassettes [9-11] have increased its suitability to be metabolically engineered. Thirdly, *Y. lipolytica* has potent production and secretion machinery for both native and heterologous proteins [12-16]. For example, wild-type strains can secrete 1 to 2 g/L of alkaline extracellular protease (XPR2) under optimal physiological conditions [17].

*Y. lipolytica* can metabolize glucose to produce lipids [18,19]. However, it cannot efficiently use lignocellulosic substrates, due to a lack of efficient cellulase and hemicellulase systems. These systems require the coordinated action of three principal types of cellulases: endoglucanase (EG), exoglucanase (cellobiohydrolase I (CBHI) and II (CBHII)), and β-D-glucosidase (BGL). Prior to the present work, EGI and most recently EGII and CBHII of *Trichoderma reesei* had been expressed in *Y. lipolytica* [20,21]. However, the expression of CBHI in *Y. lipolytica*, the most critical cellulase enzyme, had not been reported.

Previously, NREL researchers have expressed *T. reesei* CBHI in *Pichia pastoris* [22] and *Aspergillus niger* var. *awamori* [23], as well as other cellulolytic enzymes in *Zymomonas mobilis* [24]. The prospect of developing *Y. lipolytica* as a consolidated bioprocessing (CBP) organism prompted us to extend the expression of cellulases in this oleaginous organism. We now seek to test the principle that *Y. lipolytica* can be engineered to function as a CBP strain by essentially reproducing the key enzymes of *T. reesei*’s cellulase system. To achieve this goal, multiple approaches were used: First we conducted a genome wide search in *Y. lipolytica* for endogenous cellulases, including BGLs. Secondly, we expressed *T. reesei*‘s CBHII and EGII in *Yarrowia*. Thirdly, we screened several cellobiohydrolases for their expression in *Yarrowia* and subsequently achieved the successful expression of a chimeric CBHI, or the Tr-Te CBHI chimera, which contains the catalytic domain from *Talaromyces emersonii* and the linker and carbohydrate-binding module (CBM) from *T. reesei*. For simplicity, Tr-Te chimeric CBHI is generally referred to as chimeric CBHI in the text. Moreover, the purified chimeric CBHI showed a remarkable specific activity similar to the native CBHI from *T. reesei.*

Furthermore, the single-gene transformants for individual EGII, CBHII, and CBHI described above permit us to study their singular and combined effectiveness in utilizing cellulosic substrates at both the *in vitro* enzymes and the *in vivo* cell consortia levels. The primary advantage of using a consortia co-culture system is that it offers more flexibility than mono-cultures of any of the individual strains for the rational design of secretome compositions. Microbial consortia (co-fermentation systems) have been studied increasingly in recent years, because they can be programmed to execute useful tasks in bioprocessing, including degrading and converting complex cellulosic biomass to biofuels or chemicals [1,2]. Moreover, synthetic microbial consortia can consist of either multiple microbial species [3-5] or single species that contain differently engineered lines of the same species (for example, *Lactobacillus plantarum*) [6]. The *Yarrowia* cell consortia system demonstrated in this study belongs to the latter single species type.

## Results and discussion

### Morphological observation of *Y. lipolytica* culture on YPD agar plate

The morphology of *Y. lipolytica* Po1g growing on the surface of yeast peptone dextrose (YPD) medium is illustrated in Figure S1 (see Additional file 1). It is noteworthy that, upon disturbing the colonies on the plate with deionized and distilled water, the colonies dispersed into floating pieces instead of a uniform suspension, suggesting a hydrophobic nature of the cell surfaces.

### Genome-wide search for endogenous cellulase including β-D-glucosidase genes in *Yarrowia*

In total, 6,447 proteins were annotated in the genome of *Y. lipolytica* v1.0 released by Génolevures on February 9, 2012, downloaded from the JGI website and accessed on 30 November 2013. (See [http://genome.jgi.doe.gov/pages/dynamicOrganismDownload.jsf?organism=Yarli1]). Based on the downloaded amino acid sequences of these proteins, we annotated the CAZy proteins in *Y. lipolytica* using the CAZymes Analysis Toolkit [25]. As summarized in Table 1, our genomic analysis revealed the existence of genes encoding six BGLs (three of them secretory), but no genes were found in the genome for the EG and CBH that are required for breakdown of cellulose to cellobiose. It is noteworthy that the total number of predicted BGLs in *Y. lipolytica* is six, higher than that in a recent characterized oleaginous fungus *Mucor circinelloides*, which was predicted to have two extracellular and one intracellular BGL [26].

**Table 1:** **List of predicted β-D-glucosidases (BGLs) in** ***Y. lipolytica*** **genome**

**Protein ID**	**Number of amino acids**	**Domains** ^**[1]**^	**SP** ^**[2]**^
YALI0B14333g	903	GH3-GH3C-Fn3-like	N
YALI0F01672g	862	GH3- PA14-GH3C-Fn3-like	N
YALI0B14289g	869	GH3-GH3C-Fn3-like	Y; 1-17
YALI0E20185g	857	GH3	N
YALI0F16027g	844	GH3-GH3C-Fn3-like	Y; 1-14
YALI0D18381g	963	GH3-GH3C-Fn3-like	Y; 1-16

**Notes:** ^[1]^Domain architecture was deciphered using BlastP and the Conserved Domains search tool [http://www.ncbi.nlm.nih.gov/Structure/cdd/wrpsb.cgi], and the Oak Ridge CAT_orthology method.

^[2]^Signal peptides (SP) were predicted by TargetP 1.1 [http://www.cbs.dtu.dk/services/TargetP/] for the (Y) presence or (N) absence of a predicted signal peptide in the protein sequence. For proteins with predicted SP, the range of amino acids for SP is given.

The listed enzymes include those of secretory type with signal peptides (SP) predicted and non-secretory (intracellular) types.

### β-D-glucosidase enzymatic activity in *Y. lipolytica*

As described above, the genome analysis predicted three extracellular and three intracellular BGLs. Here, we further tested the BGL enzymatic activity in the supernatant as the crude enzyme. The analysis results revealed that the BGL activity peaked by 120 h culture, releasing 0.39 ± 0.02 mg glucose in the above reaction mixture, which is equivalent to 10% of substrate cellobiose being hydrolyzed into glucose within the first hour of hydrolysis.

### Expression of endoglucanase II and cellobiohydrolase II in *Y. lipolytica*: Western blot analysis

The above data indicates that the studied strain has the genes and enzymatic activity for BGL, but lacks those for EGs and CBHs. As the initial step to engineer *Y. lipolytica* for conversion to a CBP organism, we transformed *Y. lipolytica* Po1g cells with constructs containing either EGII or CBHII genes, as outlined in Table 2. Both genes were successfully transformed into *Y. lipolytica* Po1g with a high transformation efficiency, about 3,000/μg DNA.

**Table 2:** **Strains and plasmids used for expressing singular cellulolytic enzymes in this study**

**Expression plasmids**	**Plasmid description (species and GenBank accession number for expressed enzyme)**	**MW kDa**	**Source**
pINA1296 (pYLSC1)	Hybrid promoter (hp4d); secretion signal (XPR2 pre- region); selection marker gene (*LEU2*)		Madzak *et al.*, 2000
pNREL101	Tr EGII (P07982) in SfiI/XbaI cut pYLSC1	42	This study
pNREL102	Tr CBHII (P07987) in SfiI/HindIII cut pYLSC1	47	This study
**CBHI constructs**			
pNREL106	Tr CBH1 (P62694) in SfiI/XbaI cut pYLSC1	52	This study
pNREL151	Chimeric CBHI (Te CBH1-Tr Linker-Tr CBM1; AAL89553 and P62694) in SfiI/Xbal cut pYLSC1	53	This study
pNREL152	Ct CBHI (CAM98448) in SfiI/Xbal cut pYLSC1	55	This study
pNREL153	Hg CBHI (CAA35159) in SfiI/Xbal cut pYLSC1	54	This study

The strain used for the enzyme expression was *Y. lipolytica* Po1g (MATa,leu2-270,ura3-302::URA3,xpr2-332,axp-2) (Madzak *et al.*, 2000) [27]. The theoretic molecular weight (MW) was calculated based on amino acid sequences (without signal peptide). Abbreviation: CBH, cellobiohydrolase; Ct, *Chaetomium thermophilum*; EG, endoglucanase; Hg, *Humicola grisea*; Te, *Talaromyces emersonii*; Tr, *T. reesei.*

**Figure 1:**
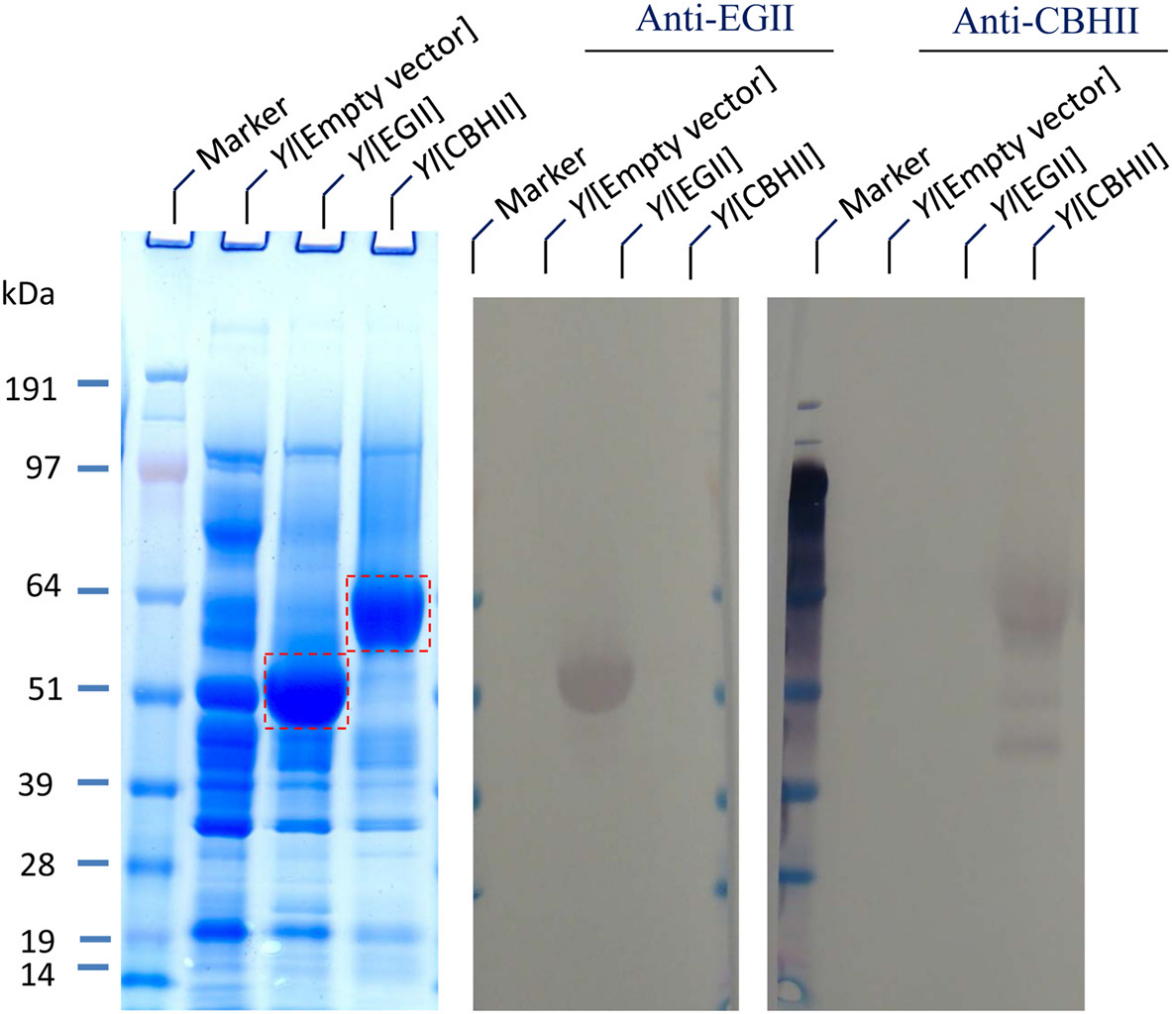
**SDS-PAGE and Western blot analyses of** ***T. reesei*** **endoglucanase II and cellobiohydrolase II expressed in** ***Y. lipolytica*** **Po1g.** The supernatants were concentrated 300-fold by ultrafiltration and concentrator. The loading amount was 20 μg proteins per well. Western blot analyses were conducted using anti-EGII and anti-CBHII antibodies, respectively. The red dotted boxes indicate the band of expressed EGII and CBHII in the PAGE-SDS gel. *Yl*, *Y. lipolytica.*

As a first step in characterizing proteins of the above *Y. lipolytica* transformants, the total secretory proteins (that is, the supernatant proteins) were extracted, separated, and analyzed by PAGE and Western blot. The results demonstrated that, compared to the empty vector transformant, the 300-fold concentrated secreted enzymes of transformants *Y. lipolytica*[EGII] and [CBHII] had extra bands in the molecular weight (MW) size of approximately 51 and 62 kDa, respectively (Figure 1). These values were slightly higher than the theoretical size of these expressed proteins (which is 42 and 47 kDa, respectively). This observation suggests that these proteins are likely to be glycosylated, and is in agreement with similar observations of expressed cellulases in *S. cerevisiae* and *P. pastoris* [28-31].

The target proteins on the SDS-PAGE gel were confirmed by Western blot analysis, using anti-EGII and CBHII antibodies, respectively (Figure 1). The amount of secreted EGII and CBHII, determined by densitometry analysis of these extra target protein bands in Figure 1, coupled with the quantitative LC/MS of cut EGII and CBHII band analysis revealed that the secretion yields for EGII and CBHII were approximately 40 mg/L and 24 mg/L, respectively in flask batch culture using YPD medium. These values are in a comparable range to the reported yield of these proteins in the recent literature [21].

It is thus reasonable to speculate that the yield of these two enzymes could significantly increase under fermentor culturing conditions, based on literature reports showing that the expression of Tr EGI in *Y. lipolytica* in fed-batch fermentation combined with high-cell density cultivation techniques has been increased 20-fold compared with the flask culture [20].

### Growth of endoglucanase II and cellobiohydrolase II *Y. lipolytica* transformants on cellulose-containing media

The transformants *Y. lipolytica*[EGII] and [CBHII] were tested for their ability to grow on cellulose plates with carboxymethyl cellulose (CMC) or Avicel cellulose as the sole substrate. EG (EC 3.2.1.4) randomly cleaves internal bonds in cellulose that generate new chain ends, as well as some glucose (which *Y. lipolytica* cells can directly utilize). Thus, it is not surprising that single colonies of transformant *Y. lipolytica*[EGII] were found to grow on CMC mineral media plates, but exhibited a filamentous morphology, which is different from the regular yeast cell type morphology on rich YPD medium. These results suggest that the cell growth is limited by carbohydrates on CMC mineral plates. Moreover, the inoculation of clusters of transformant *Y. lipolytica*[EGII] cells on CMC mineral medium and CMC YPD medium plates generated clear halo zones after Congo red staining, indicating EGII activity.

For the transformant *Y. lipolytica*[CBHII], the cells were tested on CMC mineral medium, and the results indicated that it generated clear zones on CMC mineral plates, suggesting that the expressed CBHII likely hydrolyzes CMC into soluble sugars. This observation was consistent with the mode of CBHII action. CBHII is an exoglucanase (EC 3.2.1.91) that cleaves two to four carbon units from the ends of the exposed chains generated by EG and produces tetrasaccharides or disaccharides, such as cellobiose [32]. The formed cellobiose can be converted to glucose by the above genome-predicted and functionally confirmed endogenous BGLs in *Y. lipolytica* cells; thus it supports cell growth.

Nevertheless, a detailed comparison of the halo diameter and colony diameter for transformants grown on CMC mineral medium plates showed a much smaller colony size of *Y. lipolytica*[CBHII] than that of *Y. lipolytica*[EGII], which suggests a lower capacity for degrading and utilizing the CMC substrate in *Y. lipolytica*[CBHII]. These data are consistent with those reported in the literature, where although the purified CBHII from *T. reesei* did not significantly reduce the viscosity of a CMC solution, it showed a low, but detectable activity using the traditional CMC reducing sugar assay [33]. The above observation is also consistent with another report that the *Lactobacillus* transformant expressing *Trichoderma koningii* CBHII produced hydrolysis halos on the Congo red-CMC plates [34]. To put this into perspective, our above results can be explained by the under-carboxylation of the cellulose chain ends, where the exoglucanases are able to act only to release short soluble dextrins, resulting in the apparent activity detected by the Congo red-CMC plate assay, and yet remain undetected by the viscosity assay (measurements more impacted by internal cleavage).

Furthermore, we found that the *Y. lipolytica*[CBHII] transformant cells alone cannot utilize Avicel as the sole carbon source. However, upon co-inoculation of *Y. lipolytica*[CBHII] and [EGII] cells on Avicel YPD medium, we noticed that the background of the whole Avicel YPD plate became clear after a 3-day incubation followed by Congo red staining, compared with the no inoculation and empty vector transformant controls, showing that jointly, CBHII and EGII act on crystalline cellulose.

### Hydrolytic activities of endoglucanase II and cellobiohydrolase II in culture supernatant

**Figure 2:**
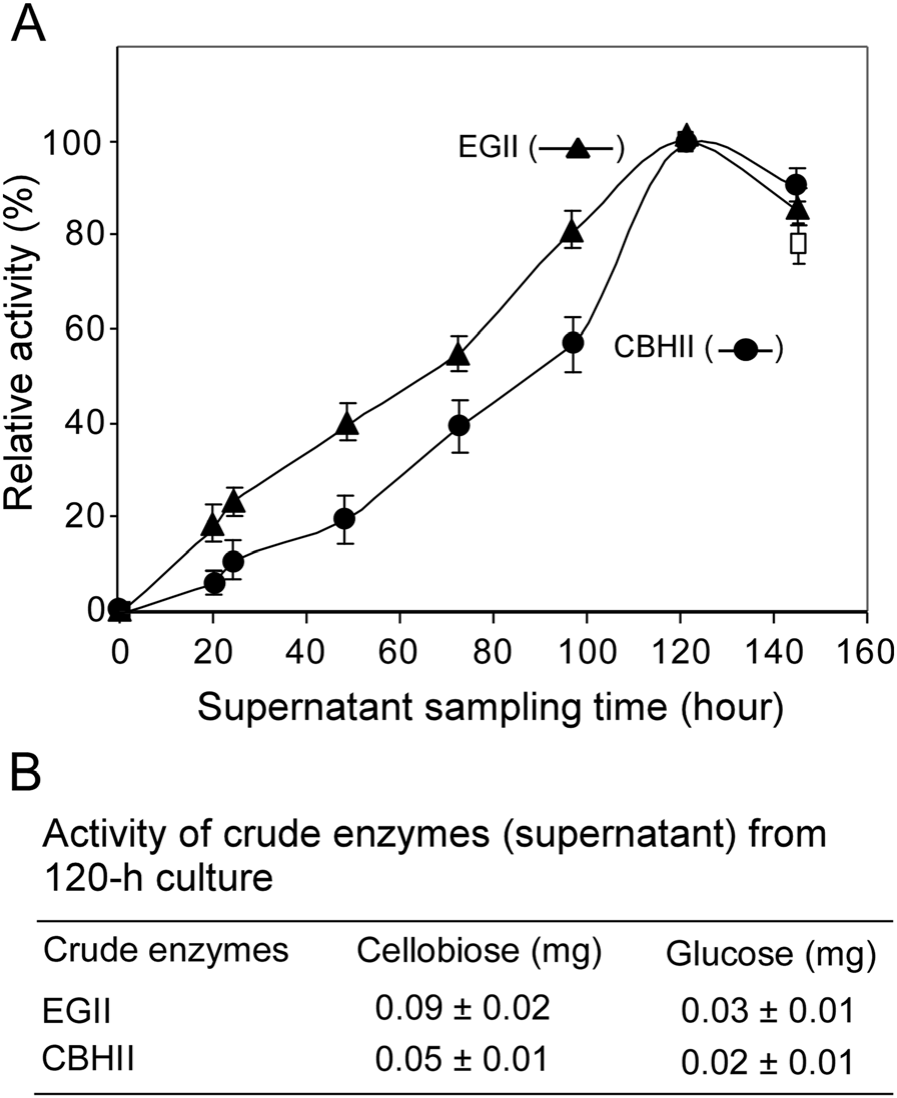
**Enzyme activity of endoglucanase II and cellobiohydrolase II in the crude enzyme preparation from the corresponding transformants grown on YPD culture.** **(A)** Culture timeline profiling of enzymatic activity in crude enzymes (that is, supernatants) collected from culture broth. **(B)** Enzymatic activity of supernatants collected at the activity peak time of 120-h cultures. Enzymatic activity assay was conducted as described in the Methods section.

To monitor the cellulase production and measure the hydrolytic activity of the cellulases produced, the *Y. lipolytica*[EGII] and [CBHII] transformants were cultured in YPD medium in flasks, and samples were taken daily. The enzyme activities of the culture broth (the supernatants) were measured by incubating the extracellular crude enzyme solution with the appropriate cellulosic substrates (phosphoric acid swollen cellulose, PASC, for EGII and Avicel for CBHII) as described in the Methods section. When the relative activities of these cellulases representing different culture sample points are graphed, their activities peak at 120 h of culture time, as shown in Figure 2A. The activities for each enzyme in the 120 h cell cultures are shown in Figure 2B, where both the *Y. lipolytica*[EGII] and [CBHII] transformants caused cellobiose and glucose release from Avicel. Note that the production of glucose can be explained by the endogenous BGL activity.

It is noteworthy that a very recent study on expressing *T. reesei* EGII and CBHII in *Y. lipolytica* also reported the use of CMC for EGII and PASC prepared from Avicel for CBHII [21]. Our study furthered the analyses in two more respects: First, both the transformant cells and their secretory enzymes were examined for plate and enzymatic assays, respectively; secondly, Avicel itself was used as the substrate in both assays. These extra aspects are needed to fulfill the eventual goal of engineering *Y. lipolytica* into a CBP organism.

In contrast to our work, the study described above for the expression of *T. reesei* EGII and CBHII in *Y. lipolytica* employed CMC for testing EGII and PASC for testing CBHII [21]. We suggest that in addition to these substrates, Avicel is probably a more suitable substrate for plate-based evaluation of a CBP strain that will be grown eventually on pretreated biomass. Specifically, the cellulosic chemical nature and recalcitrance of Avicel are closer to biomass than those of CMC and PASC, and thus are more relevant to the evaluation of the cellulolytic recombinant yeast cells. Meanwhile, in our study, both the transformant cells and their secreted enzymes were examined using plate and enzymatic assays, respectively, whereas only the secreted enzymes were tested in the previous study [21].

### Expression of *T. reesei* cellobiohydrolase I in *Yarrowia*

**Figure 3:**
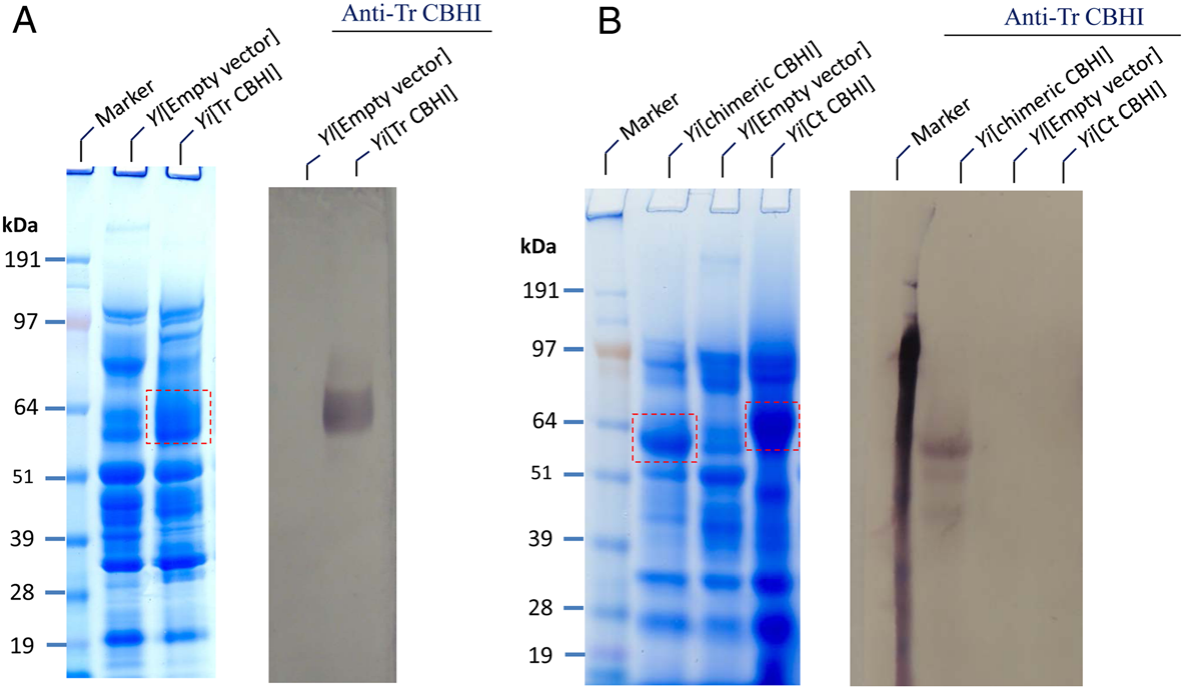
**SDS-PAGE and Western blot characterization of** ***Y. lipolytica*** **transformants expressing the** ***T. reesei*** **,** ***T. reesei-Talaromyces emersonii*** **chimeric, and** ***Chaetomium thermophilum*** **.** **(A)** Transformant expressing the *T. reesei* CBHI. **(B)** Transformant expressing the *T. reesei-Talaromyces emersonii* chimeric and *C. thermophilum* CBHIs. The supernatants were concentrated 300-fold and the loading amount was 20 μg proteins per well. Western blot analysis was conducted using anti-Tr CBHI antibody. The red dotted boxes indicate the band of expressed CBHI. Ct, *Chaetomium thermophilum*; Tr, *Trichoderma reesei*, *Yl*, *Y. lipolytica.*

The *Y. lipolytica* strain Po1g was also successfully transformed with a construct containing the Tr CBHI gene as outlined in Table 2. The total secretory proteins of the transformants were extracted, separated, and analyzed by PAGE and Western blot. The results indicated that compared to the empty vector transformant, samples prepared from the 300-fold concentrated secreted enzymes of *Y. lipolytica*[Tr CBHI] transformant had an extra band in the MW size of approximately 63 kDa (Figure 3A), which was higher than the theoretical size (52 kDa) of this protein, suggesting that it is likely to be glycosylated. The location of Tr CBHI on the SDS-PAGE gel was confirmed by Western blot analysis, using anti-CBHI antibody (see Figure 3A).

The *Y. lipolytica*[Tr CBHI] transformants did not produce conventional clear zones when grown on Avicel YPD plate. However, examination of the plate 2 h after Congo red staining and destaining revealed a deep dark zone around the colony of the *Y. lipolytica*[Tr CBHI] transformant, whereas no such zone formed around the empty vector transformant cells. The test for the *Y. lipolytica*[Tr CBHI] transformant revealed that no clear zone was observed when it grew on CMC mineral or Avicel mineral plates.

Previously, work by our group [22,35] and others [29,36,37] had demonstrated that CBHI enzymes are notoriously difficult to express in most heterologous hosts, such as *P. pastoris* and *S. cerevisiae*. Our study supports the above observation, suggesting that the folding and/or post-translation modification of heterologous CBHI in *Y. lipolytica* cannot completely mimic that in the original source strain, *T. reesei*. Considering the unique role and high efficiency of CBHI in hydrolyzing cellulose, further screening and expression of other CBHI enzymes from a variety of cellulolytic fungal species was conducted.

### Expression of cellobiohydrolases I from different sources in *Y. lipolytica*

A recent study has shown that three specific CBHIs from different cellulolytic fungal species were successfully expressed in another yeast, *S. cerevisiae*, and that their enzymatic activities were found to be less affected by glycosylation [28]. Thus, these three CBHI enzymes were chosen for expression testing in *Y. lipolytica*. These constructs included: 1) Tr-Te chimeric CBHI containing the catalytic domain from *Talaromyces emersonii* and the linker and CBM from *T. reesei*, 2) Ct CBHI from *Chaetomium thermophilum*, and 3) Hg CBHI from *Humicola grisea*.

The obtained *Y. lipolytica* transformants expressing the above CBHI constructs were first characterized using PAGE analysis of their total secretory proteins. A Western blot using the anti-Tr CBHI antibody, which recognized the CBM and linker of Tr CBHI and the chimeric CBHI, revealed a single band of a size in the range expected for the chimeric CBHI, but not in Ct CBHI (Figure 3B), confirming that the chimeric CBHI was expressed successfully. The densitometry coupled with LC/MS quantitative analysis of the chimeric CBHI band estimated that the yield of chimeric CBHI was about 130 mg/L from the fermentor culture, higher than the estimated yield of 32 mg/L from its flask culture.

Furthermore, inoculations of the chimeric *Y. lipolytica*[CBHI], [Ct CBHI], and [Hg CBHI] transformant cells on Avicel YPD medium plate all generated clear zones after Congo red staining, indicating active expression and secretion of CBHI enzymes in these CBHI transformants.

### Crude enzyme activity of chimeric cellobiohydrolase I

To measure the hydrolytic activity of the Tr-Te chimeric CBHI produced in *Yarrowia*, the supernatants of its transformant were concentrated 35-fold, and the resultant concentrated crude enzymes were incubated with Avicel substrate as described in the Methods section. As shown in Table 3, the release of cellobiose and glucose after 2 h incubation was 0.021 and 0.008 mg/mL in the saccharification mixture, respectively. With 24 h incubation, the release of cellobiose and glucose increased to 0.112 and 0.036 mg/mL, respectively.

**Table 3:** **Activities of concentrated crude enzyme of Tr-Te chimeric CBHI on releasing sugars from Avicel**

**Incubation with crude enzyme**	**Reducing sugars (mg/mL) released in saccharification mixture**	
	**Cellobiose**	**Glucose**
2 h incubation with chimeric CBHI	0.021 ± 0.002	0.008 ± 0.001
24 h incubation with chimeric CBHI	0.112 ± 0.010	0.036 ± 0.004

The sugar values illustrated are the sugar content in “enzyme plus Avicel substrate” minus “enzyme blank”. The sample of concentrated crude enzymes of chimeric CBHI was prepared by concentrating the 4-day fermentor culture 35-fold, using ultrafiltration. Values are presented as the mean (±SE) of three replicates.

### Purification and specific activity of chimeric cellobiohydrolase I

**Figure 4:**
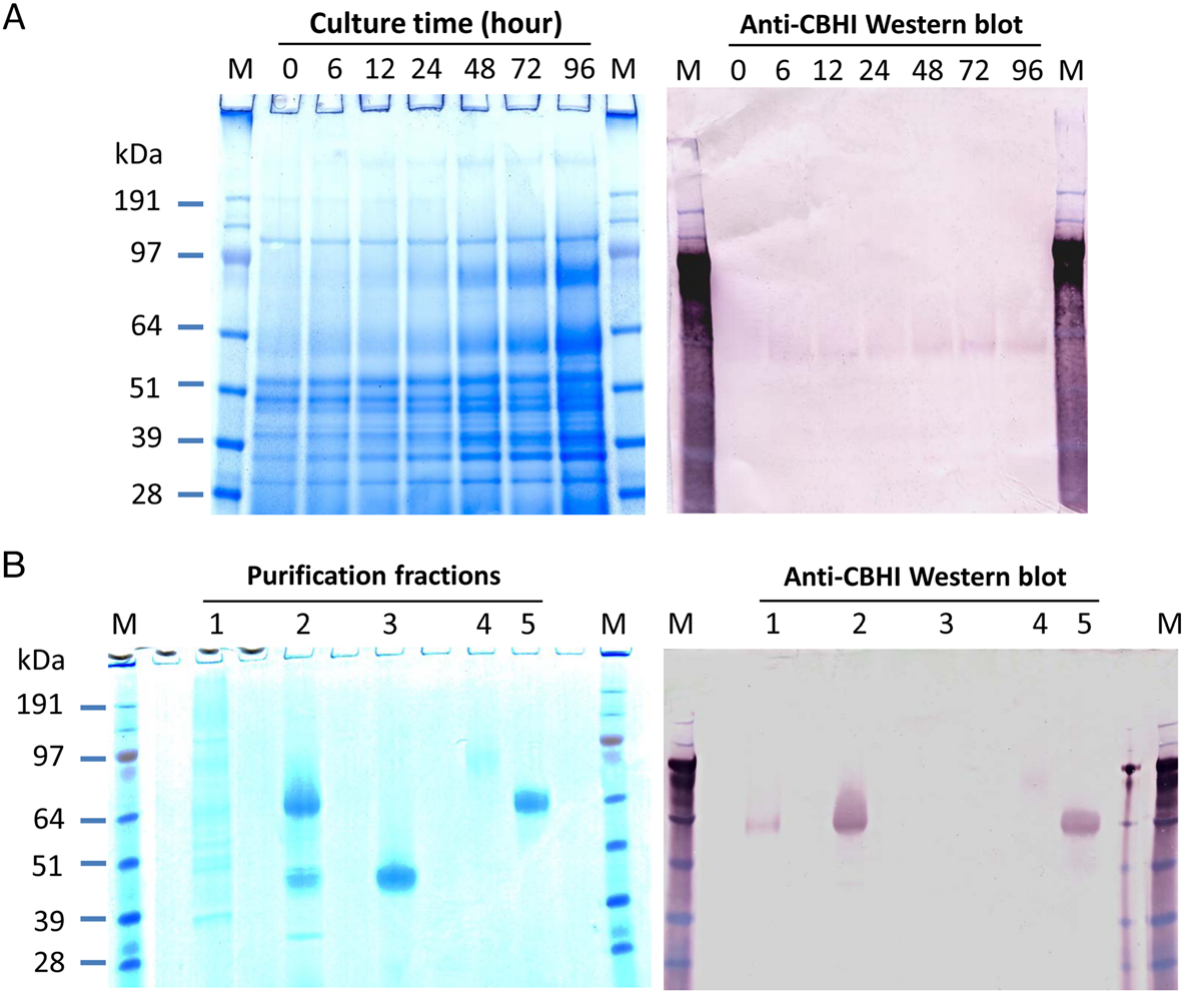
**Expression profile and purification of chimeric CBHI protein in bioreactor fermentation of** ***Y. lipolytica*** **transformant.** **(A)** Time course for the concentrated culture supernatant of *Y. lipolytica*[chimeric CBHI] transformant. The 25-fold concentrated supernatants (16 μL per well) were separated by SDS-PAGE, followed by Western blot analysis for the detection of chimeric CBHI. **(B)** SDS-PAGE and Western blot analyses for collected fractions during chimeric protein purification procedure. The chromatography fractions corresponding to gel lane numbers are: lane 1, the 20-fold concentrated culture supernatant (10 μL); lane 2, effluent from hydrophobic interaction chromatography (20 μL); lane 3, effluent from anion exchange column (20 μL); lanes 4 and 5, the minor impurity and peaks from size exclusion column (20 μL), respectively.

**Figure 5:**
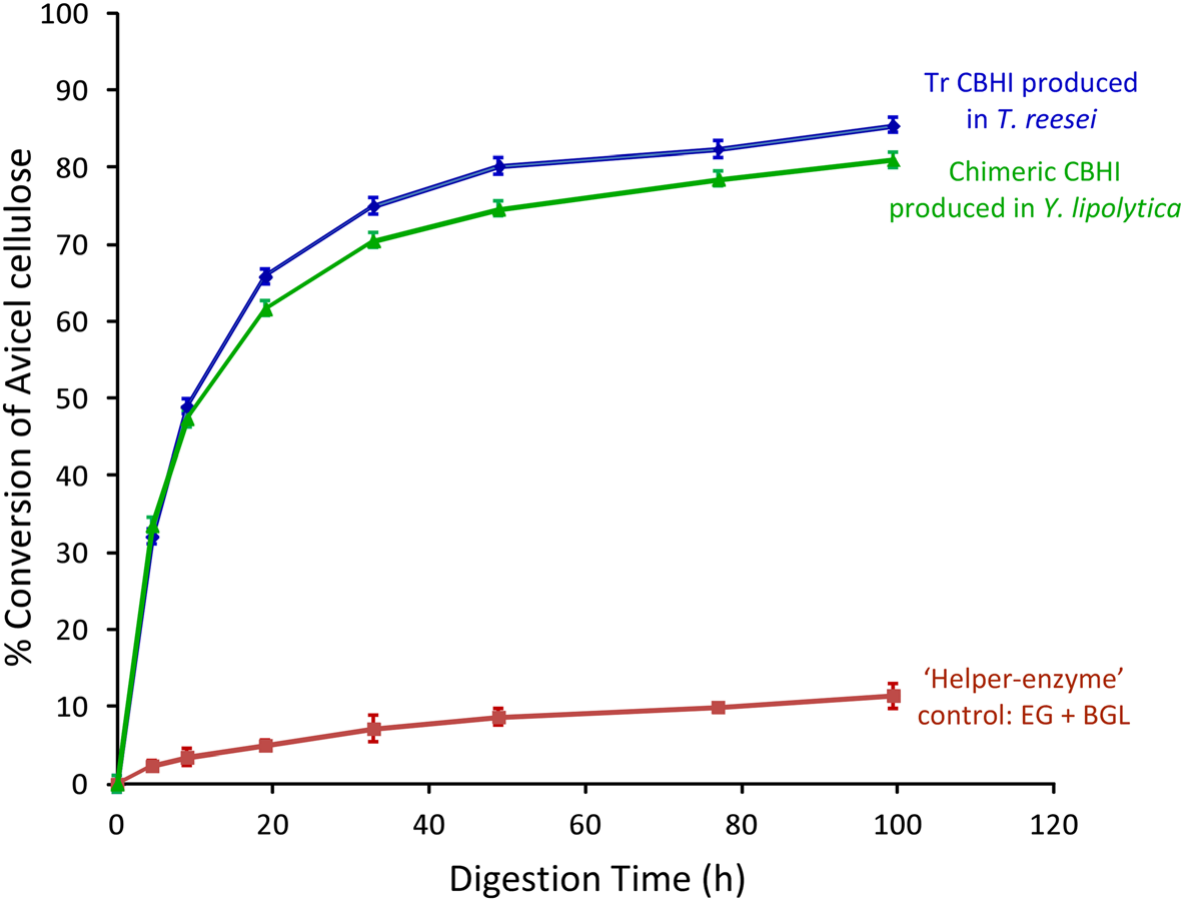
**Specific Avicelase activity of purified Tr-Te chimeric cellobiohydrolase I (CBHI).** Both chimeric and native CBHI were loaded at 46.7 mg/g cellulose, whereas the “helper enzyme” endoglucanase (EG) from *Acidothermus cellulolyticus* (that is, E1-CAT) was loaded at 1.9 mg/g cellulose, and β-D-glucosidase (BGL) at 2 mg/g cellulose. Substrate is Avicel PH-101 at 5.0 mg/mL, digestion at 40°C and pH 5.0 in 20 mM acetate. Values are presented as the mean (±SE) of three replicates.

In addition to measurement of the activities of concentrated crude enzymes, the expressed Tr-Te chimeric CBHI enzyme in *Y. lipolytica* was also purified and characterized. Its time course of production in culture and protein purification confirmation are shown in Figure 4. The final purified chimeric CBHI fraction from the 5-L 4-day fermentor culture was 0.6 mL, and it contained 6 μg/μL purified CBHI, which was used for specific activity assay. Remarkably, the purified chimeric CBHI protein showed a specific activity closely comparable to that of the native CBHI from *T. reesei* (Figure 5).

Note that although a similar chimera has been expressed in the yeast *S. cerevisiae* [28], it was expressed with its native signal peptide, and its specific activity was not reported. In contrast, the transformant expressing the chimeric CBHI in this study used the *Y. lipolytica* XPR2 signal peptide. Further tests with different signal peptides may lead to further optimization for enhancing the expression level.

### Deglycosylation analysis of chimeric cellobiohydrolase I

**Figure 6:**
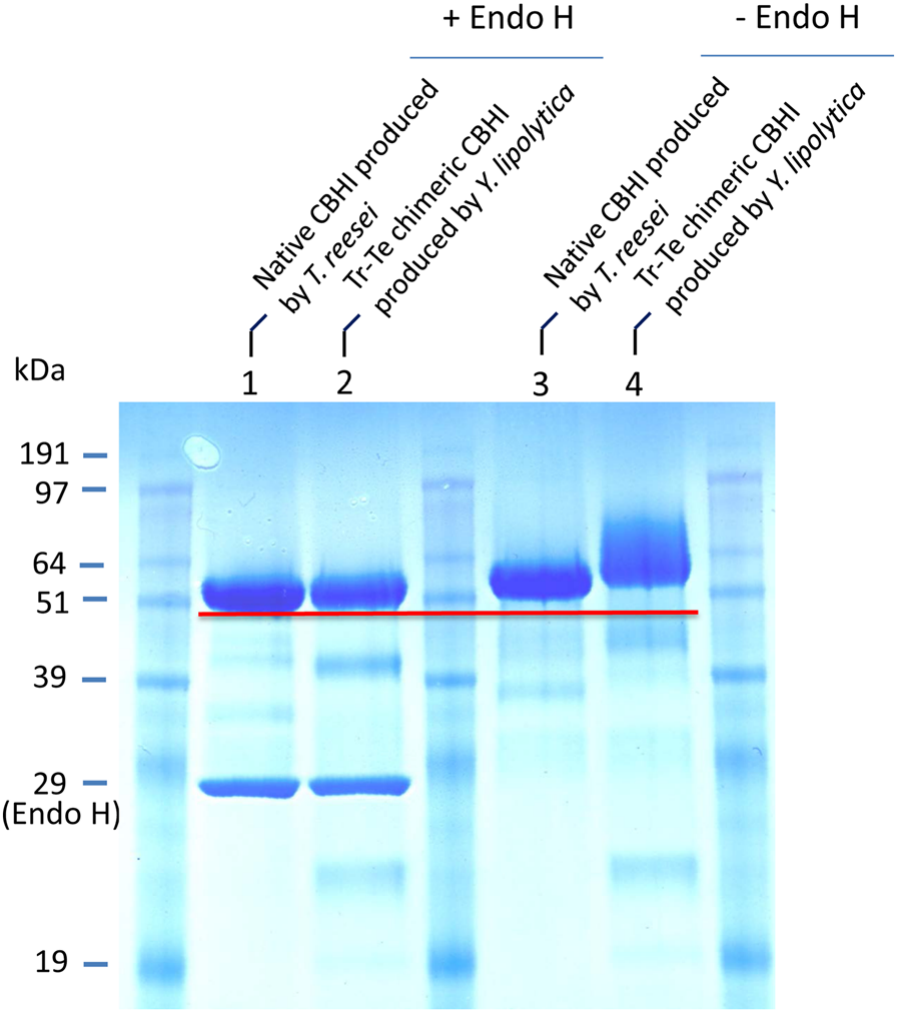
**SDS-PAGE of deglycosylated chimeric CBHI expressed in** ***Y. lipolytica*** **and native CBHI produced in** ***T. reesei*** **Rut-C30 as control.** Protein samples were deglycosylated with Endo H or non-treated (-Endo H). The protein loading was 20 μL (containing 10 μg protein) per well.

The purified chimeric CBHI protein was subjected to deglycosylation analysis to determine glycosylation and the size of the N-linked sugar chain. The purified CBHI protein was digested with endoglycosidase H (Endo H), which cleaved the N-linked oligosaccharide chain in the glycoprotein. The MW difference between protein samples with and without Endo H treatment reflects the extent of protein glycosylation mediated by the expression host. The results demonstrate that for the Tr-Te chimeric CBHI expressed in *Yarrowia*, its MW on SDS-PAGE was approximately 63 kDa for the glycosylated form (without Endo H treatment; Figure 6, lane 4) and 53 kDa for the deglycosylated form (with Endo H treatment; Figure 6, lane 2), suggesting that about 10 kDa of N-linked glycan was removed. Similarly, for native CBHI produced in *T. reesei*, its MW on SDS-PAGE was about 61 kDa for the glycosylated form and 52 kDa for the deglycosylated form, suggesting that 9 kDa of N-linked glycan was removed (Figure 6, lane 3 versus lane 1). This observation suggested that in the case of Tr-Te chimeric CBHI expressed from *Yarrowia*, the overall magnitude of glycosylation was similar or only slightly higher than that for native CBHI produced in *T. reesei*, which could partially explain why the specific Avicelase activities of these two CBHI were nearly the same.

It is noteworthy to report that after Endo H treatment, the MW from SDS-PAGE for native CBHI produced in *T. reesei* (52 kDa; Figure 6, lane 1) and Tr-Te chimeric CBHI expressed in *Y. lipolytica* (53 kDa; Figure 6, lane 2) appear to be the same as their theoretic MW calculated from their amino acid sequences, suggesting that the Endo H treatment seems to have removed most of the glycan groups that may attach to the proteins.

### Avicel cellulose utilization by co-cultures of chimeric cellobiohydrolase I, cellobiohydrolase II, and endoglucanase II producing transformants

The capability of *Y. lipolytica* cellulase transformants to utilize cellulose was evaluated by growing the transformants in the mineral medium with Avicel as the carbon source, as a mono-culture of *Y. lipolytica*[chimeric CBHI], a bi-culture of *Y. lipolytica*[chimeric CBHI] and [EGII], and a tri-culture of *Y. lipolytica*[chimeric CBHI], [EGII], and [CBHII], respectively. These cultures were harvested at 120 h, and differences in the appearance of the precipitate at the bottom of the cultures were observed. For the culture inoculated with empty vector transformants, the Avicel and cell precipitate seemed compact, whereas the precipitates of the various cultures of *Y. lipolytica*[chimeric CBHI] transformant appeared swollen and fluffy - possibly indicating changes in the cellulose caused by the transformants (Additional file 1: Figure S2 A). Microscopic imaging further revealed the changes. Cellulose fibers with sharpened tips were found in the mono-, bi-, and tri-cultures of *Y. lipolytica*[chimeric CBHI] transformant (Additional file 1: Figure S2 C to E), which is thought to be the typical effect of CBHI as recently reported [38]. Moreover, compared to the empty vector control, the particles of Avicel cellulose in the *Y. lipolytica*[chimeric CBHI] cultures were increasingly finer, showing a clearly increasing degree of synergy from bi- to tri-cultures for the decomposition of the cellulose (Additional file 1: Figure S2 C to E).

The Avicel residues from the culture were also analyzed using the cellulase digestion method described in the Methods section. The analysis results of the harvested Avicel-yeast cell pellets are summarized in Table 4. While the mono-culture of the *Y. lipolytica*[chimeric CBHI] transformant consumed 12.0% of the original Avicel content and produced 0.23 g DW of cells per g Avicel consumed, these parameters increased to 20.7% and 0.29 g for the bi-culture with *Y. lipolytica*[EGII], and to 23.5% and 0.32 g for the tri-culture with the *Y. lipolytica*[EGII] and [CBHII] transformants. Overall, the data demonstrated an increasing degree of synergy between the *Y. lipolytica*[chimeric CBHI] and [EGII] as well as the [CBHII] transformants.

**Table 4:** **Co-culturing of** ***Y. lipolytica*** **transformants expressing heterologous cellulases in 150 mL mineral medium containing 4 g Avicel (equivalent to 2.7% w/v) as sole carbon source**

**Culture of transformants**	**DW of Avicel-cell pellet**	**Avicell consumed%** ^**[1]**^	**DW of cells** ^**[2]**^	**Cell mass yield**	**Total FAME** ^**[3]**^
***Y. lipolytica*** **[enzyme]**	**g**		**g**	**g DW of cells per g Avicel consumed**	**mg in whole pellet**
[empty vector] control	4.02	0	0.02	0	N/A
[chimeric CBHI]	3.63	12.0	0.11	0.23	16.3
[chimeric CBHI] + [EGII]	3.41	20.7	0.24	0.29	24.1
[chimeric CBHI] + [EGII] + [CBHII]	3.36	23.5	0.30	0.32	28.2

**Notes:** ^[1]^Avicel degradation% was calculated as (Initial Avicel amount minus Avicel residue amount in Avicel-cell pellet) / Initial Avicel amount. The initial Avicel amount was 4 g, and the Avicel residue amount in the Avicel-cell pellet was estimated by the enzymatic hydrolysis method described in Methods section.

^[2]^Dry weight (DW) of cells was calculated as DW of Avicel-cell pellets minus Avicel residue amount in Avicel-cell pellet, in which the Avicel residue amount in the Avicel-cell pellet was measured above.

^[3]^The total FAME (fatty acid methyl esters) in the culture of *Y. lipolytica*[empty vector] could not be reliably measured due to the low amount of cell mass in the Avicel-cell pellet. N/A, not available.

Data presented are the average of duplicate samples of 120-h cultures as described in Methods section.

Furthermore, fatty acid methyl esters (FAME) analysis showed that the total FAME yield in 150 mL Avicel-mineral medium is 16.3 mg, 24.1 mg, and 28.2 mg (equivalent to 109 mg, 160 mg, and 188 mg per L culture) for *Y. lipolytica*[chimeric CBHI]’s mono-culture, the bi-culture with *Y. lipolytica*[EGII], and the tri-culture with *Y. lipolytica*[EGII] and [CBHII], respectively (Table 4). These data further confirm the synergy between the chimeric CBHI, EGII, and CBHII.

It is challenging to monitor the growth rate of the above transformant cells in Avicel-mineral medium. First, the presence of Avicel particles (with a size of about 50 μm) interferes with the OD measurement of the culture. Secondly, the current method of measuring cell mass by subtracting the Avicel residue weight (determined by the aforementioned enzymatic digestion procedure) from the weight of the cell-Avicel pellet, is time-consuming. Further study and method development are needed to efficiently quantitate the cell growth in liquid medium containing solid substrates. Although progress toward this aspect of the work will help provide deeper insights into the cellulolytic process by the cell consortia of the *Yarrowia* transformants, development of these assays is beyond the scope of this paper.

## Conclusions

In summary, we successfully screened and demonstrated the efficient secretion and function of a heterologous Tr-Te chimeric CBHI protein, along with the secretion and function of *T. reesei* EGII and CBHII, indicating their compatibility with *Y. lipolytica* as an expression host. To our knowledge, this is the first report of successful expression of CBHI in *Y. Lipolytica*. The purified Tr-Te chimeric CBHI protein showed a remarkable level of specific Avicelase activity which was indeed comparable to that of native CBHI produced by *T. reesei*, despite the fact that the deglycosylation analysis revealed that the Tr-Te chimeric CBHI protein had a slightly higher degree of glycosylation. Overall, our results strongly suggest that *Y. lipolytica* is capable of supporting the expression and secretion of high levels of core cellulases essential to biofuel production, thereby serving as a foundation for developing *Y. lipolytica* into a cellulolytic, oleaginous CBP platform organism. Future studies will be focused on combinational expression of multiple heterologous cellulases in transgenic *Yarrowia*, and on increasing the secretion, stability, and function of these cellulases.

## Methods

### Microorganisms and vectors

The *Y. lipolytica* strain Po1g (MatA, leu2-270, ura3-302:URA3, xpr2-332, axp-2) and secretion vector pYLSC1 were purchased from Yeastern Biotech Co. (Taipei, Taiwan). The *Y. lipolytica* secretion vector (pYLSC1, 7205 bp) contains the hybrid promoter (hp4d) and a secretion signal (XPR2 pre-region): atgaagctcgctaccgcctttactattctcacggccgttctggcc, which encoded signal peptide MKLATAFTILTAVLA. This vector also contains a leucine selection marker gene (*LEU2*), which can complement the deletion of the *LEU2* gene in the parental strain of Po1g.

### Constructs for heterologous cellulase gene expression

**Figure 7:**
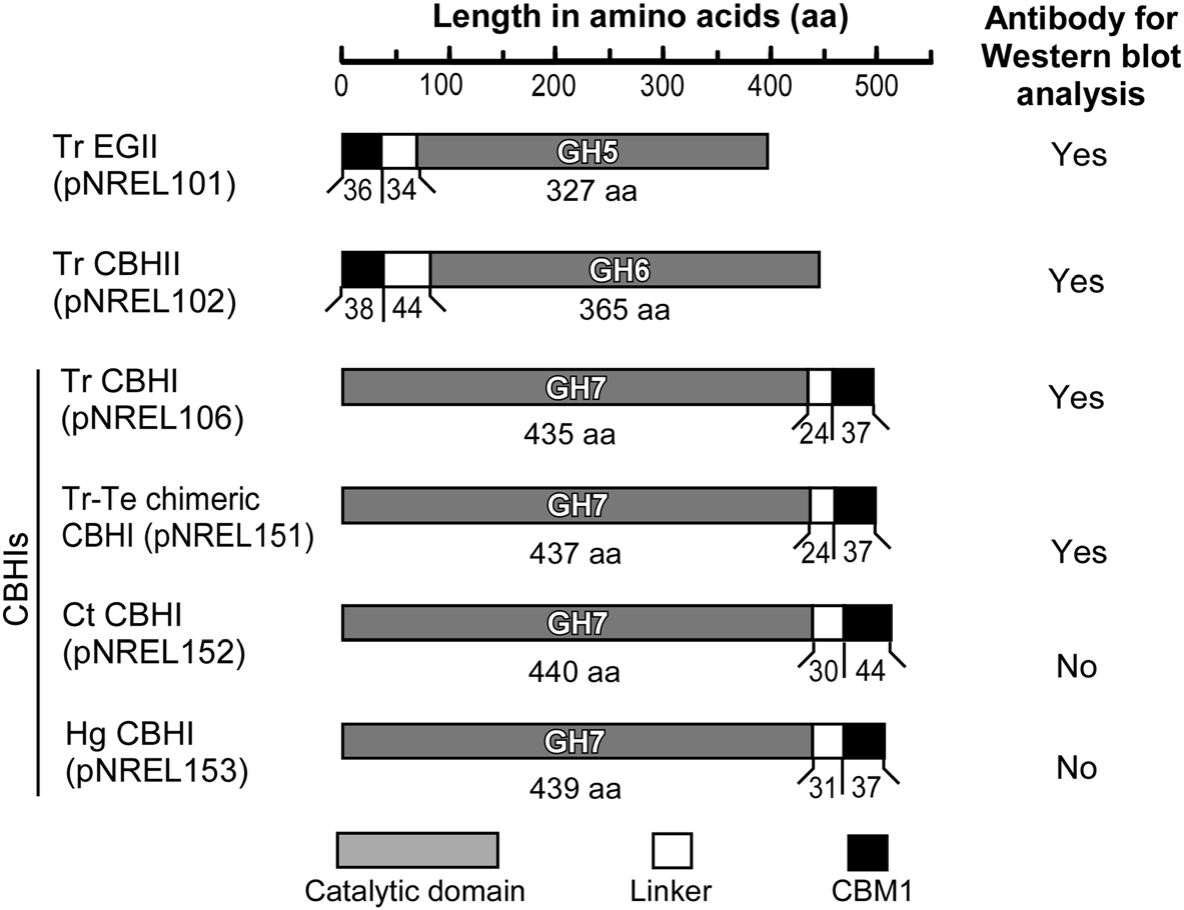
**Comparison of domain architecture of endoglucanase (EG) and cellobiohydrolases (CBHs) expressed in** ***Y. lipolytica*** **and antibody availability for Western blot analysis.** The functional domains were assigned by using the annotation information of individual proteins in UniProt and CAZy databases.

Four constructs were built in the backbone of secretion vector pYLSC1 with the hp4d promoter. The gene coding sequences of these constructs were codon-optimized based on the codon bias of *Y. lipolytica,* and were synthesized by GenScript. Table 2 summarizes the expressed cellulase genes and their corresponding *Y. lipolytica* transformants. Figure 7 illustrates the arrangement of the catalytic and CBM1 domains, as well as the linkers between them in EGII, CBHII, and multiple CBHIs. Note that Tr EGII and Tr CBHII are referred to as EGII and CBHII hereafter, whereas the various CBHI varieties are referred to in full as Tr CBH1, Tr-Te chimeric CBHI (or chimeric CBHI), Ct CBHI, and Hg CBHI for clarity in distinguishing them from each other. For the constructs of Tr CBHI, Tr-Te chimeric CBHI, Ct CBHI, and Hg CBHI, their codon-optimized nucleotide sequences with related restriction enzyme site at the 5’ and 3’ ends can be found in Additional file 1.

### SDS-PAGE and Western blot analysis

Invitrogen NuPAGE Novex 4-12% Bis-Tris Mini Gel was used for running SDS-PAGE, for which SeeBlue Plus2 Prestained Protein Standards (LC5925; Invitrogen, NY) were used as the markers. Twenty micrograms of protein concentrate was loaded into each well. Before loading onto the gel, the proteins were denatured by addition of a reducing agent (which included dithiothreitol, DTT) and heating to 70°C for 10 min. Each gel was run at 200 V constant for 35 min. After the electrophoresis, the gel was fixed with acetic acid/methanol solution, stained with colloidal Coomassie blue overnight, and destained with deionized, distilled water for 7 h.

The availability of antibodies recognizing four of the six expressed cellulases, as shown in Figure 7, makes it possible to confirm the expression of these enzymes in host cells using Western blot analysis. The gels for Western blot analysis and colloidal Coomassie blue staining were run in parallel, using the Invitrogen XCell SureLock Mini-Cell electrophoresis system and iBlot Gel Transfer System. For CBHI’s Western blot, first 4 μL of monoclonal anti-CBHI antibody in 5 mL SuperBlock T20 phosphate-buffered saline (PBS; pH 7.4) blocking buffer was applied, followed by 4 μL secondary anti-mouse antibody in 5 mL SuperBlock T20 PBS blocking buffer. For Western blots of CBHII enzymes, first 4 μL of a mono anti-CBHII antibody in 5 mL SuperBlock T20 PBS blocking buffer was applied to the blot, followed by 4 μL of secondary anti-mouse antibody in 5 mL SuperBlock T20 PBS blocking buffer. For Western blots of EGII enzymes, first 4 μL poly anti-EGII antibody in 5 mL SuperBlock T20 PBS blocking buffer was added to the blot, followed by 4 μL secondary anti-rabbit antibody in 5 mL SuperBlock T20 PBS blocking buffer. All the secondary antibodies were conjugated with alkaline phosphatase, and this activity was detected using NBT/BCIP substrates (Invitrogen).

### Growth of transformants on cellulose-containing plates

The capacity of *Y. lipolytica* transformants to degrade and utilize cellulose was assessed by growth on carboxymethyl cellulose (CMC)- or Avicel-containing agar plates on the basis of mineral medium or YPD medium. The recipe for CMC or Avicel mineral medium was modified from the literature (using KH_2_PO_4_ instead of K_2_HPO_4_) [26], whereas that for CMC or Avicel YPD medium was as follows: 1.0% yeast extract, 2.0% peptone, 2.0% dextrose, 1.5% agar. The plates were inoculated with strains, then incubated at 28°C for 3 to 5 days before Congo red staining.

### Enzyme activity assays for concentrated or unconcentrated crude enzymes

For the parental strain Po1g, the BGL enzymatic activity was measured by using the supernatant as crude enzyme. One-half milliliter of supernatant of parental strain Po1g was incubated with 0.5 mL of 1.0% w/v cellobiose (in 50 mM pH 4.8 citrate buffer) at 50°C for 1 h, and then the reducing sugars were quantified by HPLC. The tested supernatant of parental strain Po1g was collected from the culture at 24, 48, 96, 120, and 144 h.

For the *Y. lipolytica***[**CBHII] and *Y. lipolytica***[**EGII] transformants, the unconcentrated crude enzymes (the supernatants) collected from the corresponding transformants grown on YPD culture were used for enzyme activity assays. Briefly, 0.5 mL of CBHII and EGII of crude enzyme were incubated with 0.5 mL of 1% Avicel and 0.5% PASC, respectively, at 50°C for 1 h. The released sugars were then measured by HPLC.

For the *Y. lipolytica***[**Tr-Te chimeric CBHI] transformant, the supernatant collected from the YPD culture was concentrated 35-fold by ultrafiltration, and 0.5 mL of the concentrated crude enzyme preparation was incubated with 0.5 mL of 1% Avicel at 50°C for 2 h and 24 h. The released sugars were measured by HPLC.

### Fermentation and purification of chimeric cellobiohydrolase I

Production of chimeric CBHI was carried out in 5 L of a 14-L BIOFLO 3000 bioreactor (New Brunswick Scientific, Edison, NJ). A seed culture was inoculated from a single colony into 50 mL of YNB medium (20 g/L glucose, 6.7 g/L yeast nitrogen base w/o amino acids) in a 250-mL flask, incubated at 28°C and 200 rpm, and then transferred after 24 h of incubation into 500 mL of fresh YPD medium (pH 5.0) in a 2.8-L baffled flask. The secondary seed culture was subsequently transferred into the 5-L YPD medium in a 14-L BIOFLO 3000 bioreactor after 36 h of incubation. The fermentation was controlled at 28°C, 200 rpm, 1VVM air, and pH 5.0. The fermentation was run over a period of 96 h. Cells in the harvested culture broth were filtered out with a glass fiber filter and concentrated using tangential flow filtration (TFF) with a 10,000 MWCO. The concentrated culture broth was buffer-exchanged into 20 mM Bis-Tris pH 6.5 in preparation for chromatographic purification.

After concentration, the sample was purified by chromatography. First the ammonium sulfate concentration of the sample was slowly adjusted to 2 M. The sample was filtered with a 0.45-μm Nalgene Rapid-Flow Bottle Top filter (Thermo Scientific Pierce Protein Biology Products, Rockford, IL, USA) and applied to a Tricorn 10/100 hydrophobic interaction chromatography column packed with Source 15Phe resin. This column was operated with a 50 mM Bis-Tris pH 6.5 mobile phase buffer and employed a descending 2 M (NH_4_)_2_SO_4_ gradient for protein elution. The crudely purified sample was then desalted in 20 mM Bis-Tris pH 6.5 buffer using two HiPrep 26/10 Desalting columns in series before loading onto a Tricorn 10/100 anion exchange column packed with Source 15Q resin. This column was operated with 20 mM Bis-Tris pH 6.5 mobile phase buffer and an increasing 0 to 300 mM NaCl gradient for sample elution. The final purified sample was obtained using gel filtration on a 26/60 Superdex 75 in 20 mM acetate pH 5.0 mobile phase buffer and 100 mM NaCl. Whenever necessary, Vivaspin 20 10 kDa concentrators were used to concentrate the samples, and the desired protein fractions were identified using para-nitrophenyl-β-lactoside assay [39,40]. All chromatography columns, resins, and concentrators were purchased from GE Healthcare (Piscataway, NJ, USA). Protein purity was assessed by SDS-PAGE and concentration was determined using a BCA protein assay kit (Thermo Scientific Pierce Protein Biology Products, Rockford, IL, USA).

### Specific activity of purified chimeric cellobiohydrolase I

Cellulase activity was measured using microcrystalline cellulose (Avicel PH-101, Fluka; Sigma-Aldrich Corp., St. Louis, MO) as substrate. CBHI enzymes were used at a standard loading ratio of 0.45 micromole per gram of cellulose and applied to Avicel PH-101 substrate at a loading of 5 mg per mL. (Because the theoretical molecular weight of the *Yarrowia*-expressed chimeric CBHI is slightly greater than that of the *Trichoderma* CBHI, an equal molar loading resulted in a loading of 47.38 mg of the chimera per g cellulose, versus 46.68 mg protein per g cellulose for the *Trichoderma* enzyme). In addition to CBHI, each assay also contained standard background loadings of two “helper” enzymes. The first was the catalytic domain of E1 from *Acidothermus cellulolyticus*, or E1-CAT, added at 1.89 mg per g cellulose to potentiate the activity of both CBHI enzymes by the well-known exo-endo synergy, thereby allowing “substantial” conversion of the Avicel cellulose at reasonable enzyme loadings in reasonable digestion times. This approach ensures that the CBHI enzymes were catalyzing the hydrolysis of truly crystalline cellulose, rather than the “much less ordered” amorphous cellulose fraction. The second helper enzyme used in this study was *A. niger* BGL, which was chromatographically purified from the commercial mixture Novozyme 188 (Novozymes North America, Franklinton, NC, USA). BGL was loaded into the reaction mixtures at a concentration of 2.0 mg/g of cellulose substrate, a loading which is sufficient to maintain cellobiose concentrations below the levels at which cellobiose inhibition of the enzymes is measurable.

Assays were carried out at 40°C in 20 mM acetate, pH 5.0 containing 0.02% (w/v) sodium azide to inhibit microbial growth. Assays were done in triplicate, in initial digestion volumes of 1.7 mL in crimp-sealed 2.0-mL HPLC vials, with constant mixing by inversion at 10 times per min in a 40°C water bath. At designated time points during the digestions, representative 0.1-mL aliquots of liquid and solids were withdrawn for analysis. The withdrawn aliquots of digestion mixture were diluted 18-fold with deionized water into sealed 2.0-mL HPLC vials, and then immersed for 10 min in a boiling water bath to terminate the enzyme reactions. The diluted digestion-mixture aliquots were then filtered (0.2-μm Acrodisc^R^, Pall Gelman) before quantification of released sugars by HPLC. The HPLC sugar analyses were carried out on a Bio-Rad (Hercules, CA) HPX-87H column operated at 65°C with 0.01 N H_2_SO_4_ (0.6 mL/min) as mobile phase in an Agilent (Santa Clara, CA) 1100 Series liquid chromatograph with refractive index detector.

### Deglycosylation analysis of chimeric cellobiohydrolase I

Endoglycosidase H (Promega, Madison, WI) was used to remove N-linked carbohydrates from purified chimeric CBHI generated from this study, as well as from native CBHI purified from *T. reesei* Rut-C30, which was used as a control. The chimeric Tr-Te CBHI or native CBHI was treated with Endo H for 18 h at 37°C according to the manufacturer’s instructions. In the parallel treatment without Endo H (that is, -Endo H), Endo H was replaced by water. Protein samples were separated by electrophoresis using Invitrogen NuPAGE Novex 12% Bis-Tris Mini Gel and visualized with colloidal Coomassie blue staining.

### Co-culture of multiple transformants grown on Avicel cellulose substrates

As described above in the Results and discussion section, the cellulase protein secretion yield varies for different *Y. lipolytica* transformants; specifically, the secretion yield for chimeric CBHI, EGII, and CBHII proteins was approximately 32, 40, and 24 mg/L, respectively, in their corresponding transformants grown on YPD medium in flasks. Thus, the protein secretion ratio for chimeric CBHI/EGII/CBHII is 4/5/3 on a mg/L basis. Such differences in secretion yields were used to design the co-culture experiments for testing the synergy of distinct transformants in utilizing Avicel.

The seed cultures for transformants expressing an empty vector, chimeric CBHI, EGII, and CBHII, were first adjusted to the same OD value, and then used to prepare the following four types of cell mixtures by adding appropriate volumes of seed cultures (cells) to 150 mL of mineral medium containing 4 g Avicel. For example, 1) for the control, add 15 mL of empty vector transformant cells, 2) for the mono-culture, add 15 mL of *Y. lipolytica*[chimeric CBHI] transformant cells, 3) for the bi-culture, add 13.8 mL of *Y. lipolytica*[chimeric CBHI] +1.2 mL of *Y. lipolytica*[EGII] transformant cells, (4) for the tri-culture, add 8.3 mL of *Y. lipolytica*[chimeric CBHI] +1.1 mL of *Y. lipolytica*[EGII] +5.6 mL of *Y. lipolytica*[CBHII] transformant cells. After normalization with the above-described protein secretion ratios, the expected (target) cellulase protein ratio in the secretome of the bi-culture is 90/10 for the chimeric CBHI/EGII proteins, whereas that for the tri-culture is 60/10/30 for the chimeric CBHI/EGII/CBHII proteins. It is noteworthy that these targeted cellulase protein ratios are comparable to the reported optimal ratios of CBH/EG and CBHI/EGII/ CBHII for the natural *T. reesei* enzyme mixture [41].

After setup of the cell-medium-Avicel mixtures, the flasks were incubated in a rotary shaker at 200 rpm and 28°C for 5 days. The culture samples were then collected for imaging and Avicel residue analyses. Duplicate flasks were run for each type of the cell mixtures.

### Measurement of Avicel residues in Avicel-yeast cell pellet

The Avicel residues from the transformant culture growing on Avicel were mixed with yeast cells after centrifugation. The collected Avicel-yeast cell pellet was freeze-dried and weighed. The amount of Avicel contained in the pellet was determined by enzymatic digestions using the Cellic CTec2 cellulase enzyme product (Novozymes, Franklin, NC). The enzymatic digestions were then performed in 50 mL of 50 mM citrate buffer, pH 4.8, containing 1% (w/v) substrate (that is, the Avicel-yeast cell mix) and 60 mg CTec2 per g substrate. The enzymatic reaction mixture contained sodium azide (0.04% w/v) to prevent microbial growth. The reaction mixtures were placed into 125-mL Erlenmeyer shake flasks and incubated at 50°C and 130 rpm for 96 h according to NREL LAP 009 [42]. No additional BGL was added. The total glucose released was then measured by HPLC and was used to calculate the corresponding amount of Avicel contained in the Avicel-yeast cell mix. In parallel, freeze-dried Avicel was put through the same procedure as a reference.

### Lipid determination

FAME analysis of cell-Avicel residue pellet was conducted by NREL’s analytical team using the procedure previously described [26].

## Additional file

Additional file 1: Figure S1. Morphology of *Y. lipolytica* Po1g on the surface of YPD medium (A) and after being disturbed using deionized and distilled water (B). **Figure S2.** Morphology of co-culturing *Y. lipolytica* transformants expressing heterologous cellulases on mineral medium containing Avicel (2.7% w/v) as sole carbohydrates. Nucleotide sequences of constructs.

## Abbreviations

BGL: β-D-glucosidase
CBH: cellobiohydrolase
Ct: *Chaetomium thermophilum*
EG: endoglucanase
Endo H: endoglycosidase H
FAME: Fatty acid methyl esters
Hg: *Humicola grisea*
hp4d: hybrid promoter derived from pXPR2
LEU2: β-isopropylmalate dehydrogenase that catalyzes the third step in the leucine biosynthesis path
MW: molecular weight
PASC: phosphoric acid swollen cellulose
PBS: phosphate-buffered saline
Te: *Talaromyces emersonii*
Tr: *Trichoderma reesei*. Tr-Te, *Trichoderma reesei-Talaromyces emersonii*
XPR2: alkaline extracellular protease
YPD: yeast peptone dextrose

## References

[1] Beopoulos A, Cescut J, Haddouche R, Uribelarrea J-L, Molina-Jouve C, Nicaud J-M (2009). *Yarrowia lipolytica* as a model for bio-oil production. Prog Lipid Res.

[2] Tai M, Stephanopoulos G (2013). Engineering the push and pull of lipid biosynthesis in oleaginous yeast *Yarrowia lipolytica* for biofuel production. Metab Eng.

[3] Blazeck J, Hill A, Liu L, Knight R, Miller J, Pan A, Otoupal P, Alper HS (2014). Harnessing *Yarrowia lipolytica* lipogenesis to create a platform for lipid and biofuel production. Nature Commun.

[4] Ratledge C, Wynn JP (2002). The biochemistry and molecular biology of lipid accumulation in oleaginous microorganisms. Adv Appl Microbiol.

[5] Barth G, Gaillardin C (1997). Physiology and genetics of the dimorphic fungus Yarrowia lipolytica. FEMS Microbiol Rev.

[6] Dujon B, Sherman D, Fischer G, Durrens P, Casaregola S, Lafontaine I, De Montigny J, Marck C, Neuvéglise C, Talla E (2004). Genome evolution in yeasts. Nature.

[7] Sherman D, Durrens P, Iragne F, Beyne E, Nikolski M, Souciet JL (2006). Genolevures complete genomes provide data and tools for comparative genomics of hemiascomycetous yeasts. Nucleic Acids Res.

[8] Chen DC, Yang BC, Kuo TT (1992). One-step transformation of yeast in stationary phase. Curr Genet.

[9] Davidow LS, Apostolakos D, O'Donnell MM, Proctor AR, Ogrydziak DM, Wing RA, Stasko I, DeZeeuw JR (1985). Integrative transformation of the yeast *Yarrowia lipolytica*. Curr Genet.

[10] Juretzek T, Le Dall MT, Mauersberger S, Gaillardin C, Barth G, Nicaud JM (2000). Vectors for gene expression and amplification in the yeast *Yarrowia lipolytica*. Yeast.

[11] Dall MT, Nicaud JM, Gaillardin C (1994). Multiple-copy integration in the yeast *Yarrowia lipolytica*. Curr Genet.

[12] Cereghino GPL, Cregg JM (1999). Applications of yeast in biotechnology: protein production and genetic analysis. Curr Opin Biotechnol.

[13] Domínguez Á, Fermiñán E, Sánchez M, González FJ, Pérez-Campo FM, García S, Herrero AB, San Vicente A, Cabello J, Prado M (2008). Non-conventional yeasts as hosts for heterologous protein production. Int Microbiol.

[14] Madzak C, Gaillardin C, Beckerich JM (2004). Heterologous protein expression and secretion in the non-conventional yeast *Yarrowia lipolytica*: a review. J Biotechnol.

[15] Müller S, Sandal T, Kamp‐Hansen P, Dalbøge H (1998). Comparison of expression systems in the yeasts *Saccharomyces cerevisiae*, *Hansenula polymorpha*, *Klyveromyces lactis.* Schizosaccharomyces pombe and Yarrowia lipolytica. Cloning of two novel promoters from Yarrowia lipolytica. Yeast.

[16] Nicaud JM, Madzak C, Broek P, Gysler C, Duboc P, Niederberger P, Gaillardin C (2002). Protein expression and secretion in the yeast *Yarrowia lipolytica*. FEMS Yeast Res.

[17] Barth G, Gaillardin C, Wolf K (1996). Yarrowia lipolytica. Nonconventional Yeasts in Biotechnology.

[18] Papanikolaou S, Chatzifragkou A, Fakas S, Galiotou‐Panayotou M, Komaitis M, Nicaud JM, Aggelis G (2009). Biosynthesis of lipids and organic acids by *Yarrowia lipolytica* strains cultivated on glucose. Eur J Lipid Sci Technol.

[19] Tsigie YA, Wang CY, Truong CT, Ju YH (2011). Lipid production from *Yarrowia lipolytica* Po1g grown in sugarcane bagasse hydrolysate. Bioresour Technol.

[20] Park CS, Chang CC, Ryu DD (2000). Expression and high-level secretion of *Trichoderma reesei* endoglucanase I in *Yarrowia lipolytica*. Appl Biochem Biotechnol.

[21] Boonvitthya N, Bozonnet S, Burapatana V, O’Donohue MJ, Chulalaksananukul W (2013). Comparison of the heterologous expression of *Trichoderma reesei* endoglucanase II and cellobiohydrolase II in the yeasts *Pichia pastoris* and *Yarrowia lipolytica*. Mol Biotechnol.

[22] Godbole S, Decker SR, Nieves RA, Adney WS, Vinzant TB, Baker JO, Thomas SR, Himmel ME (1999). Cloning and expression of *Trichoderma reesei* cellobiohydrolase I in *Pichia pastoris*. Biotechnol Prog.

[23] Jeoh T, Ishizawa CI, Davis MF, Himmel ME, Adney WS, Johnson DK (2007). Cellulase digestibility of pretreated biomass is limited by cellulose accessibility. Biotechnol Bioeng.

[24] Linger JG, Adney WS, Darzins A (2010). Heterologous expression and extracellular secretion of cellulolytic enzymes by *Zymomonas mobilis*. Appl Environ Microbiol.

[25] Park BH, Karpinets TV, Syed MH, Leuze MR, Uberbacher EC (2010). CAZymes Analysis Toolkit (CAT): Web service for searching and analyzing carbohydrate-active enzymes in a newly sequenced organism using CAZy database. Glycobiology.

[26] Wei H, Wang W, Yarbrough JM, Baker JO, Laurens L, Van Wychen S, Chen X, Taylor LE, Xu Q, Himmel ME (2013). Genomic, proteomic, and biochemical analyses of oleaginous *Mucor circinelloides*: Evaluating its capability in utilizing cellulolytic substrates for lipid production. PLoS One.

[27] Madzak C, Treton B, Blanchin-Roland S (2000). Strong hybrid promoters and integrative expression/secretion vectors for quasi-constitutive expression of heterologous proteins in the yeast *Yarrowia lipolytica*. J Mol Microbiol Biotechnol.

[28] Ilmén M, Den Haan R, Brevnova E, McBride J, Wiswall E, Froehlich A, Koivula A, Voutilainen SP, Siika-aho M, La Grange DC (2011). High level secretion of cellobiohydrolases by *Saccharomyces cerevisiae*. Biotechnol Biofuels.

[29] Boer H, Teeri TT, Koivula A (2000). Characterization of *Trichoderma reesei* cellobiohydrolase Cel7A secreted from *Pichia pastoris* using two different promoters. Biotechnol Bioeng.

[30] Saloheimo M, Lehtovaara P, Penttilä M, Teeri T, Ståhlberg J, Johansson G, Pettersson G, Claeyssens M, Tomme P, Knowles J (1988). EGIII, a new endoglucanase from *Trichoderma reesei*: the characterization of both gene and enzyme. Gene.

[31] Teeri TT, Lehtovaara P, Kauppinen S, Salovuori I, Knowles J (1987). Homologous domains in *Trichoderma reesei* cellulolytic enzymes: gene sequence and expression of cellobiohydrolase II. Gene.

[32] Garver MP, Liu S, Gupta VG, Tuohy M, Kubicek CP, Saddler J, Xu F (2014). Development of thermochemical and biochemical technologies for biorefineries. Bioenergy Research: Advances and Applications.

[33] Irwin DC, Spezio M, Walker LP, Wilson DB (1993). Activity studies of eight purified cellulases: specificity, synergism, and binding domain effects. Biotechnol Bioeng.

[34] Zhao Y **Expression and activities of recombinant CBHII gene in*****Escherichia coli*****and*****Lactobacillus.*** MSc thesis. Jilin Agricultural University, Faculty of Animal Science and Technology; 2008.

[35] Jeoh T, Michener W, Himmel ME, Decker SR, Adney WS: **Implications of cellobiohydrolase glycosylation for use in biomass conversion.** *Biotechnol Biofuels* 2008, **1:** 10.1186/1754-6834-1-10 PMC2427024 18471276

[36] Penttilä ME, André L, Lehtovaara P, Bailey M, Teeri TT, Knowles JK (1988). Efficient secretion of two fungal cellobiohydrolases by *Saccharomyces cerevisiae*. Gene.

[37] Van Arsdell JN, Kwok S, Schweickart VL, Ladner MB, Gelfand DH, Innis MA (1987). Cloning, characterization, and expression in *Saccharomyces cerevisiae* of endoglucanase I from *Trichoderma reesei*. Nature Biotechnol.

[38] Brunecky R, Alahuhta M, Xu Q, Donohoe BS, Crowley MF, Kataeva IA, Yang S-J, Resch MG, Adams MW, Lunin VV (2013). Revealing nature’s cellulase diversity: the digestion mechanism of *Caldicellulosiruptor bescii* CelA. Science.

[39] Tabatabai M, Bremner J (1969). Use of *p*-nitrophenyl phosphate for assay of soil phosphatase activity. Soil Biol Biochem.

[40] Van Tilbeurgh H, Claeyssens M (1985). Detection and differentiation of cellulase components using low molecular mass fluorogenic substrates. FEBS Lett.

[41] Kallioinen A, Puranen T, Siika-aho M (2014). Mixtures of thermostable enzymes show high performance in biomass saccharification. Appl Biochem Biotechnol.

[42] Brown L, Torget R **Enzymatic saccharification of lignocellulosic biomass. Laboratory Analytical Procedure 009.** National Renewable Energy Laboratory (NREL); 1996 [http://www.nrel.gov/biomass/pdfs/42629.pdf]

