# Supplementary material for: Engineering towards a complete heterologous cellulase secretome in Yarrowia lipolytica reveals its potential for consolidated bioprocessing
Source: Biotechnol Biofuels. 2014 Oct 16;7:148. doi: 10.1186/s13068-014-0148-0 (PMC4203959; doi:10.1186/s13068-014-0148-0)
Supplement: Additional file 1: Figure S1. — Morphology of Y. lipolytica Po1g on the surface of YPD medium (A) and after being disturbed using deionized and distilled water (B). Figure S2. Morphology of co-culturing Y. lipolytica transformants expressing heterologous cellulases on mineral medium containing Avicel (2.7% w/v) as sole carbohydrates. Nucleotide sequences of constructs. [file 13068_2014_148_MOESM1_ESM.docx]

## Engineering towards a Complete Heterologous Cellulase Secretome in *Yarrowia lipolytica* Reveals Its Potential for Consolidated Bioprocessing

**Additional file 1**

List of contents:

**Supplementary Figure S1.** Morphology of *Y. lipolytica* Po1g on the surface of YPD medium (A) and after being disturbed using deionized and distilled water (B).

**Supplementary Figure S2.** Morphology of co-culturing *Y. lipolytica* transformants expressing heterologous cellulases on mineral medium containing Avicel (2.7% w/v) as sole carbohydrates.

**Nucleotide sequences of constructs.**


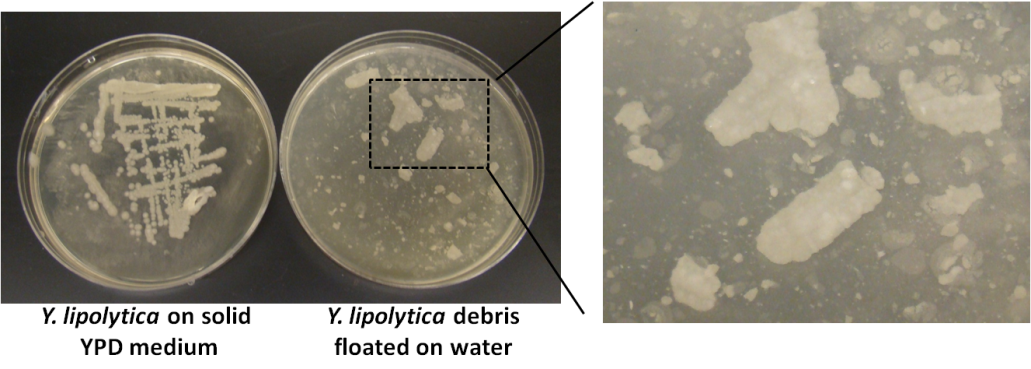


**Supplementary Figure S1.** Morphology of *Y. lipolytica* Po1g on the surface of YPD medium (A) and after being disturbed using deionized and distilled water (B).

**
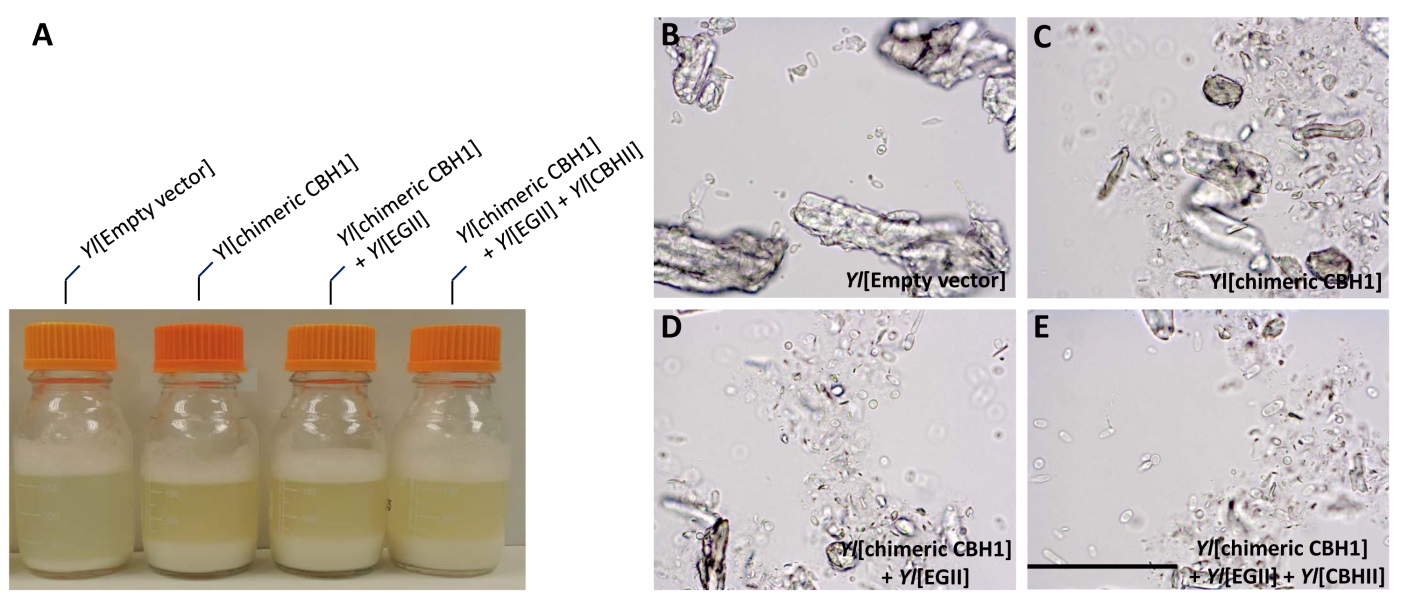
**

**Supplementary Figure S2.** Morphology of co-culturing *Y. lipolytica* transformants expressing heterologous cellulases on mineral medium containing Avicel (2.7% w/v) as sole carbohydrates. **(A)** Settling-down pattern of Avicel cellulose residues after being utilized by *Y. lipolytica* transformants. The initial culture of *Y. lipolytica* transformants was conducted in baffled flasks for 120 h, then transferred to PYREX 250-mL round bottle for 20 min before picture was taken. (**B-D**) Morphological comparison of Avicel granules and the yeast cells for *Y. lipolytica* transformants, which include *Y. lipolytica*[empty vector] (B), *Y. lipolytica*[chimeric CBHI] (C), *Y. lipolytica*[chimeric CBH1] + *Y. lipolytica*[[EGII] (D), *Y. lipolytica*[chimeric CBH1] + *Y. lipolytica*[[EGII] + *Y. lipolytica*[CBHII] (E). The scale bar represents 75 µm. *Yl*, *Y. lipolytica.*

**Nucleotide sequences of constructs.**

Note that these nucleotide sequences were codon-optimized based on the codon bias of *Y. lipolytica*, and are started and ended with related restriction enzyme sites for cloning into the vactor of pYLSC1 (see the Methods section for details).

>pNREL106_construct_Tr_CBH1

GGCCGTTCTGGCCGGCCGTTCTGGCCCAGAGCGCTTGCACTCTGCAGTCTGAAACCCACCCTCCCCTCACCTGGCAGAAGTGTAGCTCAGGAGGTACGTGTACCCAGCAAACTGGCAGTGTTGTTATTGACGCTAACTGGCGATGGACTCACGCCACCAACTCTTCCACCAACTGCTACGATGGTAACACTTGGTCGTCTACCCTCTGTCCCGATAACGAGACTTGTGCCAAAAATTGCTGTCTCGACGGTGCCGCATACGCCTCCACCTATGGTGTCACCACATCTGGTAACTCGCTGTCCATCGGCTTCGTGACCCAGTCAGCCCAGAAAAACGTCGGAGCTAGACTTTACCTTATGGCGTCAGACACTACCTATCAAGAGTTCACCCTCCTGGGAAACGAGTTCTCCTTCGACGTTGATGTCAGTCAGCTGCCTTGTGGACTGAACGGAGCCTTGTACTTCGTGTCGATGGACGCTGATGGAGGAGTATCCAAGTACCCCACGAACACAGCCGGAGCTAAGTACGGCACTGGCTACTGTGACTCGCAGTGCCCTCGTGACCTTAAGTTCATTAACGGACAGGCTAATGTCGAGGGCTGGGAGCCTTCCTCCAACAATGCCAACACCGGAATCGGTGGTCACGGCTCATGCTGTAGCGAGATGGACATCTGGGAGGCCAATTCCATTTCCGAGGCCCTGACCCCGCATCCCTGCACAACCGTTGGCCAGGAAATCTGCGAGGGCGACGGATGCGGAGGAACTTACTCTGACAACCGGTACGGTGGGACGTGTGATCCTGATGGCTGCGATTGGAACCCCTATCGACTGGGAAACACCTCTTTTTACGGTCCGGGATCGTCTTTCACCTTGGACACAACGAAAAAGCTCACAGTCGTTACGCAGTTTGAGACTTCGGGTGCCATCAACCGGTATTACGTGCAGAACGGCGTGACTTTCCAGCAACCGAACGCAGAGCTAGGTTCGTACAGCGGGAACGAACTCAACGATGACTATTGTACCGCTGAGGAGGCCGAGTTTGGCGGCTCCAGCTTTTCTGATAAGGGTGGCCTCACCCAGTTCAAAAAGGCTACCTCAGGCGGCATGGTGTTGGTTATGAGCCTGTGGGACGACTACTACGCGAACATGCTGTGGCTCGACTCAACGTACCCCACCAATGAAACATCTTCTACCCCTGGCGCTGTGCGAGGCTCGTGCTCGACTTCCTCCGGTGTCCCAGCACAAGTGGAATCTCAGTCTCCTAATGCTAAGGTCACCTTTTCCAACATTAAGTTCGGTCCTATCGGGTCTACCGGTAACCCCTCTGGAGGAAATCCCCCAGGTGGCAACCGAGGCACGACAACTACACGCCGTCCCGCAACTACGACCGGCAGTTCTCCCGGACCCACGCAGTCGCATTACGGTCAGTGCGGAGGCATTGGTTACTCGGGACCTACCGTGTGCGCTAGTGGAACTACTTGTCAGGTGCTTAACCCTTACTACTCCCAATGTCTGTGATAATCTAGAATGATAATCTAGAA

>pNREL151_construct_Chimeric_CBHI

GGCCGTTCTGGCCCAGCAGGCAGGGACCGCTACCGCCGAGAACCATCCTCCCCTCACCTGGCAGGAGTGCACCGCACCCGGATCCTGCACCACGCAGAACGGCGCTGTCGTGCTGGACGCAAATTGGCGATGGGTTCACGATGTGAACGGGTACACTAACTGCTATACAGGCAACACATGGGATCCTACCTACTGCCCCGACGACGAAACATGTGCCCAGAACTGTGCCCTTGACGGAGCTGACTACGAGGGAACCTACGGGGTCACCTCGTCCGGGAGCAGTCTCAAGCTCAACTTTGTGACCGGTTCAAACGTCGGTTCACGGCTCTATCTGCTCCAGGACGACTCGACCTACCAGATCTTCAAACTGTTGAACCGAGAGTTCTCGTTTGACGTTGATGTTTCGAACTTGCCTTGCGGACTTAATGGTGCTCTGTACTTTGTTGCAATGGATGCTGACGGCGGAGTCTCTAAGTACCCCAACAACAAGGCTGGTGCCAAGTACGGTACGGGCTATTGTGACAGTCAGTGCCCTAGAGATCTAAAATTCATTGATGGCGAGGCCAACGTCGAGGGCTGGCAACCGAGCAGCAATAACGCCAATACTGGAATCGGCGACCACGGCTCCTGTTGCGCGGAGATGGACGTGTGGGAAGCGAACTCCATTAGTAATGCTGTAACACCCCATCCGTGCGACACTCCGGGACAGACGATGTGTTCCGGTGACGATTGTGGCGGCACCTACTCCAACGATCGATACGCGGGTACATGCGATCCCGACGGCTGCGATTTCAACCCATACCGGATGGGTAATACATCCTTCTATGGACCAGGTAAGATTATCGACACTACGAAGCCTTTCACCGTGGTCACGCAGTTTTTGACAGATGACGGCACTGATACCGGAACCCTTTCGGAAATCAAGCGCTTTTACATCCAGAACTCTAATGTTATTCCCCAACCCAACAGTGACATTTCCGGAGTTACTGGAAACTCTATCACTACAGAGTTTTGTACAGCCCAAAAGCAGGCCTTCGGGGACACCGACGACTTTTCTCAGCACGGAGGCCTGGCCAAAATGGGAGCTGCCATGCAACAGGGAATGGTGCTTGTCATGTCTCTGTGGGATGACTACGCCGCTCAAATGCTGTGGCTGGACTCTGACTACCCCACTGATGCCGATCCAACTACCCCTGGCATCGCCAGAGGTACTTGTCCCACCGACAGCGGCGTGCCGTCCGACGTAGAGTCCCAGTCGCCCAACAGCTATGTGACGTACTCGAACATTAAGTTCGGCCCTATCAACTCTACGTTCACCGCATCTAACCCACCTGGTGGTAACCGAGGTACTACGACCACTCGACGTCCTGCTACTACCACTGGTTCGTCACCCGGACCCACCCAGTCTCACTACGGACAGTGTGGCGGAATTGGATACTCTGGTCCCACCGTGTGTGCGTCTGGAACTACCTGTCAGGTCCTCAACCCTTACTACTCCCAATGCCTGTGATAATCTAGAA

>pNREL152_construct_Ct_CBHI

GGCCGTTCTGGCCCAGCAGGCTTGCTCCCTTACAGCCGAAAATCACCCCTCTCTGACGTGGAAGCGGTGTACCAGTGGGGGTAGCTGTTCTACTGTGAACGGAGCCGTCACTATTGATGCTAACTGGAGATGGACCCACACCGTTTCGGGCAGTACGAACTGTTATACCGGAAACCAGTGGGACACCTCTTTGTGCACCGACGGCAAGTCTTGCGCACAGACATGTTGTGTTGACGGGGCAGACTACTCGTCCACCTACGGTATCACGACTTCGGGCGACAGCCTCAACCTGAAGTTTGTGACAAAGCATCAGTACGGCACTAATGTGGGGTCACGAGTGTATCTCATGGAGAATGACACGAAGTATCAGATGTTCGAGCTGCTAGGTAATGAGTTCACTTTTGATGTCGATGTGTCTAACCTCGGCTGCGGCCTGAACGGTGCTCTGTACTTTGTTTCTATGGACGCGGACGGAGGAATGTCTAAGTACTCGGGAAACAAGGCAGGAGCCAAATACGGCACTGGATACTGTGACGCCCAGTGCCCCAGGGATCTGAAGTTTATCAACGGCGAGGCCAACGTCGGTAATTGGACGCCTAGTACTAATGACGCCAACGCCGGTTTCGGACGATACGGTTCCTGCTGTAGCGAGATGGACGTGTGGGAAGCTAACAACATGGCGACCGCGTTTACTCCACATCCCTGCACAACCGTTGGACAGTCCCGATGTGAGGCCGACACGTGTGGTGGCACCTACTCGTCGGACCGCTACGCGGGAGTCTGTGATCCCGATGGCTGCGACTTCAACGCCTACAGACAAGGAGACAAGACCTTCTACGGCAAGGGAATGACCGTCGATACAAACAAAAAGATGACCGTGGTGACACAGTTCCACAAAAATTCTGCCGGTGTCCTTTCCGAGATTAAGCGGTTCTACGTTCAGGACGGAAAGATCATCGCCAACGCTGAATCAAAGATCCCGGGAAATCCCGGCAACTCAATTACTCAGGAATACTGCGACGCTCAAAAGGTTGCCTTTTCCAACACTGATGATTTCAACCGTAAAGGAGGAATGGCCCAGATGTCCAAGGCTCTGGCCGGTCCTATGGTCCTGGTGATGTCTGTGTGGGACGATCACTACGCAAACATGCTCTGGCTCGACAGCACTTATCCAATCGATCAAGCTGGCGCTCCTGGCGCAGAGCGTGGTGCGTGTCCGACAACCAGCGGAGTGCCCGCTGAGATTGAGGCTCAAGTACCAAACTCGAACGTCATTTTCTCTAACATTCGATTTGGCCCCATCGGCTCAACCGTCCCTGGATTGGACGGTTCCAACCCCGGAAACCCCACCACCACAGTAGTGCCCCCTGCCTCCACGTCCACTTCCCGACCTACTTCGTCTACGTCCTCACCTGTTTCTACCCCGACCGGTCAGCCTGGAGGTTGCACCACCCAAAAGTGGGGCCAGTGTGGCGGGATTGGCTACACCGGTTGCACAAACTGCGTCGCTGGCACAACTTGCACCCAGTTGAACCCCTGGTATAGTCAGTGTCTTTGATAATCTAGAA

>pNREL153_construct_Hg_CBHI

GGCCGTTCTGGCCCAACAAGCCTGTTCGCTGACCACTGAACGACATCCTTCTCTGTCCTGGAACAAGTGCACCGCTGGAGGACAGTGTCAGACCGTTCAGGCGTCTATCACCCTCGACTCGAACTGGCGTTGGACTCATCAGGTGTCAGGTTCTACCAATTGCTACACCGGCAACAAGTGGGATACCTCGATCTGCACTGACGCTAAGTCATGCGCACAGAACTGCTGTGTGGACGGAGCCGACTACACCTCGACCTACGGAATCACCACAAACGGGGACAGCCTTAGTCTCAAATTTGTAACTAAGGGCCAGCACTCCACGAATGTGGGCTCCCGAACCTACCTTATGGACGGGGAGGACAAGTATCAAACATTCGAGCTTCTGGGAAACGAGTTCACATTCGATGTGGATGTCTCGAACATTGGATGCGGCTTGAACGGTGCACTGTACTTTGTCTCGATGGATGCTGACGGTGGCCTGTCTCGATACCCCGGTAACAAGGCTGGAGCGAAGTACGGTACTGGCTATTGCGACGCCCAGTGTCCTAGAGACATTAAGTTCATCAACGGCGAAGCAAACATTGAGGGATGGACTGGCTCAACCAACGACCCCAACGCCGGAGCAGGCAGATACGGAACATGTTGTTCCGAGATGGATATTTGGGAGGCCAACAATATGGCAACAGCTTTCACGCCCCACCCCTGCACTATTATCGGTCAAAGCCGGTGTGAGGGTGACTCCTGTGGCGGCACCTACTCCAACGAGCGCTACGCCGGTGTTTGTGACCCCGATGGTTGTGATTTTAACTCTTACCGTCAGGGTAACAAGACCTTCTACGGCAAGGGCATGACTGTCGATACCACTAAAAAGATTACTGTGGTTACCCAGTTTCTCAAAGACGCCAATGGAGATCTGGGCGAGATTAAGCGATTCTACGTGCAGGACGGCAAGATTATCCCTAACTCTGAATCTACCATTCCAGGCGTCGAAGGTAATTCGATCACTCAAGACTGGTGTGACAGGCAGAAGGTCGCGTTTGGCGACATCGACGATTTCAACCGGAAAGGAGGCATGAAACAGATGGGCAAGGCCCTCGCTGGCCCTATGGTCTTGGTTATGAGCATTTGGGACGACCACGCGTCTAACATGCTGTGGCTGGACAGCACGTTTCCCGTGGATGCTGCCGGAAAGCCCGGAGCTGAGAGAGGGGCCTGTCCCACGACGTCCGGAGTGCCCGCTGAGGTCGAGGCTGAAGCCCCTAACAGTAACGTTGTGTTTTCCAACATTCGATTCGGTCCGATCGGGTCCACGGTTGCCGGACTACCTGGAGCTGGAAACGGAGGAAACAATGGTGGCAACCCGCCTCCGCCAACAACAACCACTTCATCTGCTCCTGCCACAACCACCACGGCGTCTGCCGGTCCCAAGGCCGGTCGATGGCAGCAGTGCGGTGGAATCGGTTTCACAGGCCCAACTCAGTGCGAAGAGCCCTATATCTGCACCAAGCTCAATGATTGGTATAGTCAGTGCCTATGATAATCTAGAA
